# Social Isolation, Loneliness and Generalized Anxiety: Implications and Associations during the COVID-19 Quarantine

**DOI:** 10.3390/brainsci11121620

**Published:** 2021-12-08

**Authors:** Linas Wilkialis, Nelson B. Rodrigues, Danielle S. Cha, Ashley Siegel, Amna Majeed, Leanna M. W. Lui, Jocelyn K. Tamura, Barjot Gill, Kayla Teopiz, Roger S. McIntyre

**Affiliations:** 1 Mood Disorders Psychopharmacology Unit, University Health Network, Toronto, ON M5T 2S8, Canada; Linas.Wilkialis@mail.utoronto.ca (L.W.); nelson.rodrigues@mail.utoronto.ca (N.B.R.); cha.danielle@gmail.com (D.S.C.); sieg8296@mylaurier.ca (A.S.); amna.majeed@mail.utoronto.ca (A.M.); leanna.lui@mail.utoronto.ca (L.M.W.L.); jocelyn.tamura@mail.utoronto.ca (J.K.T.); barjotgill@gmail.com (B.G.); kayla.teopiz@mail.utoronto.ca (K.T.); 2 Institute of Medical Science, University of Toronto, Toronto, ON M5S 1A8, Canada; 3 Department of Pharmacology, University of Toronto, Toronto, ON M5S 1A8, Canada; 4 Department of Psychiatry, University of Toronto, Toronto, ON M5T 1R8, Canada; 5 Brain and Cognition Discovery Foundation, Toronto, ON M4W 3W4, Canada

**Keywords:** COVID-19 pandemic, implications, social isolation, loneliness, mechanism, solutions

## Abstract

The COVID-19 pandemic has resulted in a predominantly global quarantine response that has been associated with social isolation, loneliness, and anxiety. The foregoing experiences have been amply documented to have profound impacts on health, morbidity, and mortality. This narrative review uses the extant neurobiological and theoretical literature to explore the association between social isolation, loneliness, and anxiety in the context of quarantine during the COVID-19 pandemic. Emerging evidence suggests that distinct health issues (e.g., a sedentary lifestyle, a diminished overall sense of well-being) are associated with social isolation and loneliness. The health implications of social isolation and loneliness during quarantine have a heterogenous and comorbid nature and, as a result, form a link to anxiety. The limbic system plays a role in fear and anxiety response; the bed nucleus of the stria terminalis, amygdala, HPA axis, hippocampus, prefrontal cortex, insula, and locus coeruleus have an impact in a prolonged anxious state. In the conclusion, possible solutions are considered and remarks are made on future areas of exploration.

## 1. Introduction

At the end of 2019, the World Health Organization (WHO) was informed of a series of emerging cases of pneumonia with unknown etiology in Wuhan, China [1]. In the following weeks, it was determined that these cases were due to a severe acute respiratory virus known as COVID-19, caused by a novel coronavirus, SARS-CoV-2 [1]. The virus rapidly spread throughout China and nearby countries and around the world, resulting in a global pandemic [1]. As with previous pandemics, quarantine, the restriction of movement of persons suspected of coming into contact with an infectious disease, has been implemented as a tool by officials to curtail the spread of the virus around the globe [2,3].

Notwithstanding the efficacy of quarantine as a tool to limit the spread of disease, it also creates social isolation. Social isolation is an objective state in which an individual is alone, and is a sufficient, but not necessary, condition to produce the subjective feeling of loneliness. More specifically, some individuals may be socially isolated but experience this as a natural and healthy state in normal conditions; however, the prolongation of social isolation, particularly in unnatural conditions (e.g., in response to a global pandemic) can become a hazardous state to individual health [4,5]. Loneliness is a similar yet distinct case: it is a subjective state in which a person feels that their social connections are inadequate (even though they may have a substantial social network). Both of these concepts relate to social connections and the maintenance of a healthy support network. Unsurprisingly, as quarantine has increasingly been used to limit the social contact of individuals during the COVID-19 pandemic, there have been emerging studies reporting the negative impact that quarantine has on both social isolation and loneliness [6,7,8,9,10].

In order to understand why these two states have a negative impact on health when prolonged under unusual circumstances, we must examine why social connections are necessary for healthy functioning. The social baseline theory proposes that we are social beings and therefore naturally require social connections to reproduce and survive [11]. When these requirements are unmet (e.g., in quarantine, which by definition limits our social interaction), psychological and physiological consequences occur in response to the restriction of our social environment [12]. This is evident in the reported morbidities and early mortalities associated with social isolation [4,5], as well as the alarming health consequences of pathological loneliness (where day-to-day activities are affected) [4,13]. Evidence seems to suggest, therefore, that when we have inadequate social support (whether objective or subjective), our body performs maladaptively, and it can be speculated that these prolonged conditions play an important role in the psychopathology of various mental disorders.

Taken together, it is unsurprising that these two conditions are exacerbated by their association with depression, anxiety, and suicide; in particular, generalized anxiety disorder is intensified by comorbid health implications [14,15,16,17]. Generalized anxiety disorder (GAD) is a major health concern [18] that is prevalent at 5% worldwide [19]. GAD affects the quality of life and further impacts life due to its comorbid nature [20,21]. GAD urgently needs to be addressed with recent reports projecting a substantial increase in prevalence due to the ongoing quarantine to mitigate the spread of COVID-19 [6,7,8,9,10,22]; this increase may have unforeseen impacts post pandemic.

**Figure 1:**
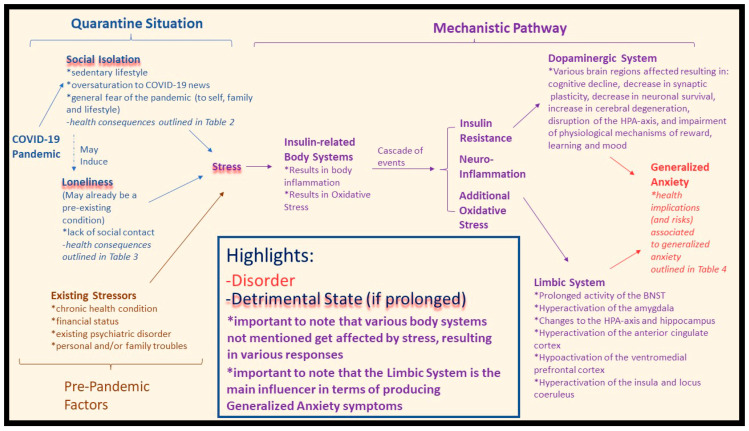
General proposed pathway influenced by various factors. Physiological concepts are applied to formulate the pathway. The proposed mechanism where the COVID-19 pandemic has caused quarantine response: social isolation conditions have a number of health issues associated with stress (highlighted in blue). Loneliness may be induced by social isolation, or make an existing loneliness condition worse, and is also associated with stress (highlighted in blue). Pre-pandemic factors also play a role (highlighted in brown). The resulting stress is associated with a cascade of events (highlighted in purple), ending with a link to generalized anxiety.

While existing literature has reported on the effects of social isolation, loneliness, and the deterioration of mental health during the COVID-19 pandemic, to our knowledge, no review has demonstrated their link to generalized anxiety. The purpose of this review is to analyse the association of social isolation and loneliness to generalized anxiety in quarantine-like conditions; acknowledging this association will be a starting point in addressing the decrease in mental wellness during the COVID-19 pandemic. This analysis includes a review of the independent morbidities of these factors and proposes a potential construct, operationalized herein as the proposed mechanism wherein the COVID-19 pandemic has engendered conditions of social isolation with significant implications on health outcomes via disparate stress responses (Figure 1), to explain their association.

## 2. Methods

The steps taken during data selection to reduce bias and accurately report the findings are described below. A narrative review was undertaken, as our aim was to provide a framework wherein quarantine as a response to COVID-19 impacted social isolation and loneliness and see how these relate to GAD.

We searched the online databases Ovid MEDLINE/Pubmed, Google Scholar, and Google Search engine (for online articles) from their inception to 8 November 2021. In choosing our data, we searched for primary articles, review articles, online articles, and books that were related to the following concepts/terms: COVID-19, social isolation, loneliness, anxiety, general anxiety, function, inflammation, insulin resistance, mechanism, pathway. These concepts/terms were chosen based on preliminary discussions on “COVID-19”, “Loneliness”, “Social Isolation” and “Quarantine”. The current, available research led us to conclude that there was a need to examine the effects of social isolation, loneliness, and the deterioration of mental health during the COVID-19 pandemic and their possible association with GAD (i.e., a series of interconnected ideas forming the basis for an updated narrative review). Manual searches were also conducted using the reference list of any relevant articles.

All work was accepted if it was available in the English language. Titles and abstracts were examined to determine if the article was relevant to this review. Once deemed relevant, the discussion section was examined for further relevance to our topic. Following the confirmation of relevance, the entire work was analysed to collect data for our review. From preliminary searches, we deemed that there were few eligibility restrictions to impose, as both the virus itself was still active (hence the research on its effects is still in its infancy) and the current studies were limited to being conducted online. Due to the focus of our review, the study population was applicable to all those affected during the pandemic (i.e., the general population), although the majority of the data focused on adults and the elderly. No restrictions were placed upon the type of measurements chosen by studies for the various terms mentioned, as outlined in Table 1.

## 3. Results

### 3.1. Social Isolation during COVID-19: Health Consequences

Quarantine is a viable tool for combating the spread of COVID-19 [3]; however, its sequela, social isolation, particularly when prolonged, increases the risk of infringing on many individuals’ needs for social interactions. Maslow’s Motivational Theory of Needs (1943) first outlined a hierarchy of human needs, which Matias et al. have adapted slightly and incorporated into the current landscape of quarantine [37]. Using a physiological and psychological perspective, they outlined how each level of “need” is specifically impacted by quarantine, resulting in a disbalance of body equilibrium [37]. The resulting behaviours are our innate drive to bring our body back into balance, and, as such, their outline will be discussed as a framework herein [37]. In keeping with this view, when our body is in disbalance due to unmet needs, certain acute health consequences result, as listed in Table 2.

The foundation level of this hierarchical structure is “Immediate Physiological Needs” (i.e., hunger, thirst, sex, elimination, sleep), which were the first to be affected by the COVID-19 pandemic [37]. Stress and anxiety caused by the ‘lockdown’ responses have created psychological conflict in individuals, where they panic buy and stockpile supplies, incidentally affecting two foundational physiological needs—food and drink. While these behaviours are undertaken in order to provide comfort through food and drink, the consequences include weight gain, obesity, and eating disorders [23,37]. Sleep is another basic foundational physiological need that is disturbed during conditions of isolation produced by the pandemic [25]. As sleep is vital for healthy body maintenance and repair, the reduction in sleep quality and/or quantity places individuals at risk for physiological deterioration and attenuated immune function [43]. Furthermore, along with its negative impact on sleep, the increased propensity toward a sedentary lifestyle has a number of physiological effects on cardiovascular, metabolic, and endocrine systems, creating further health implications (Table 2) [40].

The “Need for Self-Protection” is the next level of basic human needs affected by the stay-at-home government policies [37]. The fear of infection and death along with unmet self protection needs among result in deep-rooted feelings in individuals in lockdown of not being able to protect themselves or their family [37]. The resulting frustration contributes to adverse health outcomes and behaviours when the need for self-protection is unmet in isolated conditions (Table 2). Recent data have demonstrated various health consequences in vulnerable individuals during COVID-19 lockdowns—conditions such as an increase in alcohol consumption (hazardous drinking increased to 29.1%, harmful drinking to 9.5%), deterioration of mental well-being (32.1% reported), various forms of anxiety (29% reported), different forms of depression (37.1% reported), and increased risk of suicide [6,35,36]. Furthermore, these factors are found to result in insomnia, irritability, and aggression (e.g., physical violence) [6,37], which is evinced by the increase in homicides and suicides during the current pandemic [37,44]. While these factors refer to individual consequences, lockdown policies result in groups of individuals spending prolonged periods of time together (e.g., roommates, families), creating unique and abnormal periods of interaction. This highlights that there are not only health implications at the individual level but at a family-dynamic level as well.

The next affected need is the “Need for Affiliation” [37]. Connecting with others is a natural need that helps an individual deal with controlling emotions, coping with stress and remaining resilient; however, quarantine policies limit this much-needed face-to-face contact. While virtual options exist, they are missing nonverbal cues, lack warmth and provide less engagement, resulting in a reduced quality of connection [34]. As a result, social isolation and loneliness increase any existing stress, which can have harmful effects on immune and cardiovascular health [37,40]. These effects include deterioration in various body functions, ranging from the neuromuscular system to energy balance and inflammation, as depicted in Table 2. Importantly, individuals who feel unfulfilled in their need for affiliation are at greater risk of failing to meet the next level of need.

The last need affected by isolation is the “Need for Status/Self-Esteem”. The pandemic has caused increased unemployment and poverty, which are related to decreased self-esteem in affected individuals [37]. This outcome leads to an increased vulnerability to depressive symptoms and an increase in alcohol consumption [37]. The previously mentioned data by Ahmed et al. suggests that 37.1% of participants affected by the pandemic-lockdown experience depressive symptomatology, and hazardous drinking has increased to 29.1%, with 32.1% of participants experiencing a decline in mental health [6].

The foregoing health concerns associated with social isolation provide an important emphasis on the inciting factors that place individuals at risk of these outcomes. Numerous stressors have been found to be associated with these social isolation outcomes, including, but not limited to, longer quarantine duration, fear of being infected, frustration, boredom, inadequate supplies, inadequate information, financial loss, and stigma associated with the illness [41]. Addressing these stressors will directly help reduce any possible health outcomes associated with social isolation and indirectly treat any behaviour/consequence related to those outcomes (e.g., alcoholism, depression, loneliness, anxiety).

As these social isolation health risks are becoming more apparent, recent studies have identified a number of vulnerable subgroups in the general population. The elderly have been identified as being more likely to suffer psychologically; however, it is suggested that this is due to the high mortality rate in this clinical population amidst the pandemic (i.e., the perception of an active threat and particular vulnerability incites mental stress) [22,23]. Furthermore, elders were found to have depressive symptomatology due to a lack of informational technology (I.T.) skills (i.e., inability to connect with others) [42]. Interestingly, elders were found to be less likely to socially isolate even thought they were at the greatest risk, which the authors concluded was due to the psychological conflict of wanting to maintain a normal lifestyle [22,23]. Nonetheless, elders are still vulnerable to social isolation risk as they are forced to adhere to quarantine guidelines (i.e., those that adhere to isolation guidelines are therefore at risk, and those that choose not to adhere to quarantine guidelines are limited in their social interactions due to the current quarantine policies). Online questionnaires found that the younger age group (ages 21–40 years) and women were more vulnerable to mental health issues, as their stress increase was associated with social media usage (i.e., more access to the oversaturation of COVID-19 related news) [22,31,45]. Social isolation is generally associated with physical inactivity, and as such, younger people are vulnerable to loneliness due to the association of physical inactivity with loneliness (e.g., adolescents being physically inactive were more likely to feel lonely) [29]. It is important to understand these physical/mental health consequences, the stressors, and groups vulnerable to the effects of social isolation, as it informs us as to how anxiety is associated with the current COVID-19 solution. Figure 1 depicts a construct in which straining conditions, such as social isolation, are associated with symptoms of anxiety during the COVID-19 pandemic.

### 3.2. Loneliness during COVID-19: Health Concerns

It is proposed that the increase in physiological stress response (e.g., increased levels of cortisol) due to the COVID-19 pandemic results in an overall abnormal stress response that negatively affects health outcomes [46]. For example, social isolation has been linked to the subjective experience of loneliness, which has been reported to have a significant impact on mental and physical health, resulting in adverse overall health outcomes, as outlined in Table 3 [13,46,47].

As discussed in a recent manuscript by Wilkialis et al. [64], loneliness is concerning as it is associated with a number of adverse health outcomes, including, but not limited to, GAD, major depressive disorder, suicide, and increased mortality [13,14,27]. Furthermore, loneliness was highly prevalent before the COVID-19 pandemic [13,59]. We theorize that the current COVID-19 pandemic and the implementation of quarantine as a public health strategy to prevent its spread is increasing the rate of loneliness. Notably, the elderly are identified as a population more vulnerable to loneliness due to their increased susceptibility to the virus and subsequent need to quarantine to minimize the spread of COVID-19 [26,47,65]. Taking this into consideration, the relationship between the COVID-19 pandemic alongside the rate and severity of loneliness is increasing, especially in people previously or currently infected, with elders being disproportionately affected [28,30]. We therefore conclude that with the implementation of social isolation (i.e., quarantine) the rate of subjective experiences of loneliness are also increasing.

Loneliness is associated with various health impacts, and it is important to highlight possible variables that place individuals at increased risk to adverse health outcomes. In addition to social isolation, there are a number of risk factors for loneliness. For example, living in a rural area, poor functional status (especially cognitive impairment), widowhood, being female, subjective causes (illness, death, etc.), depression, feeling misunderstood by others, and living alone (quarantine/social isolation for our purposes) [49]. Indeed, a recent study reported similar risk factors associated with high rates of loneliness during the COVID-19 pandemic: being female, being younger, having fewer family resources (less contact with relatives), having fewer personal resources, and having a negative self-perception of aging [7]. The main concern is that because of the current COVID-19 pandemic, loneliness is becoming an increasing and/or worsening problem [46]; this will have many acute and long term effects on individuals and may lead to other illnesses that have already been associated with loneliness (e.g., GAD) [13,66].

### 3.3. Social Isolation, Loneliness, and Generalized Anxiety during COVID-19

Taken together, the implication of both social isolation and loneliness allows us to highlight their differences and distinct negative outcomes on both physical and mental health. Nonetheless, it is important to acknowledge that both conditions have been intertwined, with their various health implications being comorbid [4,15,16]. The foregoing observations, along with research suggesting that social isolation, because of the pandemic, has led to increased loneliness [28,30], provide the impetus to analyze their combined effects on both physical and mental health. These combined effects consist of a wide array of risk factors that have both a direct and indirect association to GAD, as outlined in Table 4. Recent online studies show evidence supporting this association by confirming the increase in self-reported symptoms of anxiety and psychological distress during the COVID-19 pandemic lockdown [6,8,9,10,24].

The independent health implications of social isolation and loneliness (Table 2 and Table 3) also play a role in the increased rate of anxiety, which is discussed herein. In terms of gender, the literature suggests that males are more prone to alcohol consumption during quarantine [6] and women are more prone to stress and higher levels of generalized anxiety pertaining to their overall health [10,22]. Elderly populations are especially vulnerable to the pandemic due to the loneliness and social isolation conditions, in which exposure to the radio, TV, and media can increase fear, generalized anxiety, and depression [22,42]. Those already suffering from loneliness and social isolation are even more affected [46]. Additional important findings to note are that people with higher education (more self aware of their health) [22], people with previous or current psychiatric illness [10,32], existing chronic illness [10], current COVID-19 patients [33] and those living in urban areas [10] are also found to be more vulnerable to the associated mental health consequences of the pandemic. We therefore theorize that these foregoing factors and their resulting effects are directly and indirectly associated with GAD. Understanding these effects, particularly in vulnerable groups, will further help elucidate the association of social isolation and loneliness with GAD during the COVID-19 pandemic.

Mounting data suggests that individuals are more prone to symptoms of generalized anxiety during the COVID-19 quarantine; increasing rates during quarantine conditions are expected, as well as more cases post pandemic. Furthermore, symptoms of generalized anxiety are associated with a number of health morbidities due to its comorbid nature, including, but not limited to, depressive disorders, substance abuse disorders, mood disorders, somatic symptom disorder, heart disease, chronic respiratory disorder and gastrointestinal conditions [19,21]. When taking these into account with the implications of GAD, social isolation, and loneliness, there is a mixture of health hazards for affected individuals. These hazards will result in acute and chronic morbidities, leading to possible premature death. To acknowledge and address this, it was noteworthy to begin by outlining the independent and combined health consequences of social isolation and loneliness and their possible association with GAD.

### 3.4. Quarantine and the Limbic System: A Mechanistic Perspective

Considering the impact that generalized anxiety may have on health secondary to the COVID-19 pandemic, it is worth exploring potential constructs to gain an understanding of how this disorder may be unfolding. The potential mechanism underlying social isolation, loneliness, and their negative health impacts on the etiology of GAD will be discussed; the Negative Valence Domain is applicable as its systems are responsible for responses to unpleasant situations or contexts, such as fear, anxiety, and loss [69]. The Negative Valence Systems involve mainly the limbic system; however, it is important to note that it is not the only system involved [70]. “NIMH » Negative Valence Systems: Workshop Proceedings”, Davis et al., and Lebow et al. provide detailed reviews of the neuroanatomy of anxiety, which will be used as a framework herein [70,71,72].

Our proposed construct of how the COVID-19 pandemic lockdown may be disrupting the above-mentioned limbic system is outlined in Figure 1. The quarantine imposed upon residents of many countries has resulted in an “epidemic” of social isolation, and has resulted in a number of maladaptive health situations, as outlined in Table 2 and Table 4, which lead either to direct stress or conditions associated with stress [40,41]. In addition, social isolation may induce a state of loneliness or exacerbate existing loneliness [46]. Regardless of whether it is existing loneliness or social isolation-“induced” loneliness, research has shown how loneliness is associated with underlying pathophysiological mechanisms, resulting in stress and stress-like conditions (abnormal states), as outlined in Table 3 and Table 4 [4,14]. It is also important to note that individuals may be experiencing pre-existing stressors that are exacerbated by the COVID-19 pandemic (i.e., from social isolation and loneliness).

At this point, it is important to keep in mind the previously mentioned Negative Valence Systems and their relevant constructs. Firstly, the five main constructs can all be applied to the COVID-19 pandemic: *responses to acute threat*—fear of catching the virus itself; *responses to potential harm*—fear of future “fallout” caused by the virus; *responses to sustained threat*—constant exposure to the “state” of the pandemic and its consequences for various months; *frustrative non-reward*—prevention of normal life; and *loss*—loss of job, loved ones, and social life due to the quarantine/virus [70]. Secondly, the limbic system comes under strain due to the accumulated stress [70].

The accumulation of psychological and pathophysiological adaptations to stress has alarming implications on the brain. As discussed in a recent manuscript by Wilkialis et al. [64], insulin-related body systems are put under strain, resulting in body-wide inflammation and oxidative stress [73,74,75]. A cascade of events follow that result in eventual insulin resistance, neuro-inflammation, and oxidative stress [73,74,76]. Available evidence demonstrates that insulin-resistance disrupts the dopamine system (among other factors), resulting in the following effects: cognitive decline, decrease in synaptic plasticity, decrease in neuronal survival, increase in cerebral degeneration, disruption of the HPA axis, and impairment of physiological mechanisms of reward, learning, and mood [74,76,77]. In terms of the limbic system, it has been suggested that inflammation alters the performance of certain regions associated with symptoms of generalized anxiety [78,79]. The bed nucleus of the stria terminalis (BNST) is a region sensitive to inflammation. When activated by corticotrophin-releasing factor (CRF), the effects are found to be long-lasting and can result in a prolonged “anxious” state [70,71,72]. The amygdala is well known to be an inflammation/stress sensitive area in which prolonged exposure causes hyper-activation [70,71,72,80]. It has been noted that inflammatory (stress) induced changes (due to prolonged exposure) to the HPA axis and hippocampus, result in anxiety-like behaviour [70,81,82,83]. The prefrontal cortex can also create adverse effects due to unwanted inflammation. The anterior cingulate cortex (ACC), when hyperactivated due to inflammatory cytokines, produces symptoms of anxiety [79,84]; the ventromedial prefrontal cortex (vmPFC) on the other hand, becomes hypoactive, meaning that it does not regulate signals coming from the amygdala as it normally would, therefore resulting in symptoms of anxiety [79,85]. Similar consequences (hyperactivation) are found in the insula and locus coeruleus due to inflammation [79,80,86]. Interestingly, a reduction in trust-associated activation of the anterior insula and medial prefrontal cortex (the neurocircuits of trust that connect to other limbic regions) was suggested to contribute to the non-trusting, anxious behaviour observed in lonely individuals [87]. Furthermore, a recent systematic review by Lam et al. has highlighted structural and functional differences in the above mentioned regions in terms of loneliness [88], suggesting that these systems may play a role in both GAD and loneliness.

**Figure 2:**
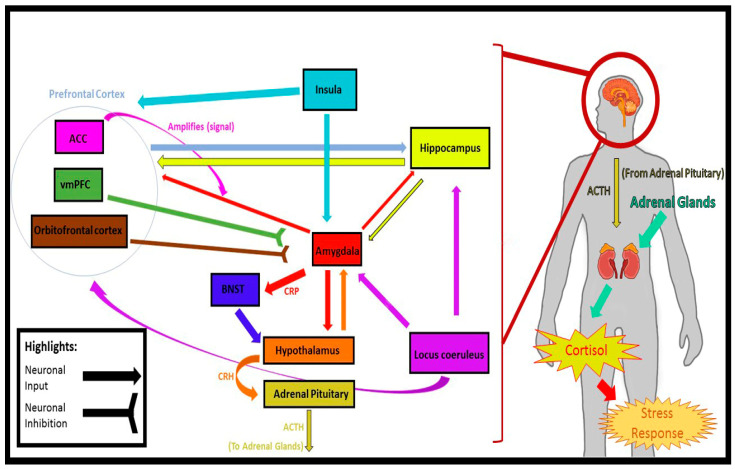
The limbic system areas and respective connections in terms of a stress response (fear/anxiety). While the limbic system is the primary source for a stress response, please note that that these are not the only brain and body systems involved. Midsagittal brain image adapted from gero.usc.edu [89] (accessed on 16 July 2020). HPA axis based on Figure 1 from Tapp et al. [81].

It is therefore logical, when considering all these variables, to arrive at the concluding proposition: that there is indeed a process taking place during the COVID-19 pandemic that is associated with GAD or anxiety-related conditions. Therefore, the construct proposed herein provides a theoretical mechanism wherein the COVID-19 pandemic has engendered conditions of social isolation with significant implications for health outcomes via disparate stress responses resulting in symptoms of anxiety (Figure 2).

## 4. Discussion

The COVID-19 pandemic has indeed created an unprecedented situation. It has impacted global trade and travel and has compelled many world governments to enforce quarantine to halt the spread of the virus [2]. Future pandemics, and the likelihood of a future wave, will undoubtedly require additional quarantine (social isolation type) enforcement. While quarantine is an appropriate solution to dealing with the virus itself, we need to address the complications that arise from social isolation, including, but not limited to, loneliness and symptoms of generalized anxiety; this includes being aware of the harm that quarantine does to vulnerable populations.

### 4.1. Future Directions: Solutions

Social isolation has a number of health consequences (Table 2), the majority of which are associated with a sedentary lifestyle. Recommended solutions to deal with these effects are to maintain a healthy diet and take daily exercise, as these help an individual keep their mental and physical state in balance [40,46], as well as to make a slight reduction to daily energy intake [40] and ensure that basic needs are met (e.g., access to food, medication, and face masks) [41,42]. For populations where exercise may be a more difficult option to access (i.e., lower SES, chronic health conditions), exercise accessibility options should be considered by policy makers.

Loneliness and its associated health outcomes (Table 3) should be addressed by aiming to maintain social connections within the limits of the prevailing social-distancing restrictions. For instance, providing I.T. assistance for elders so that they can stay connected with family and friends by using online platforms [42,46]. For all ages, promoting the use of online social connections by video call (not just texting) to satisfy individual social connection needs [26,41]. Creating support groups that help people feel needed and provide them with any aid/advice that they may require during quarantine [59,90]. It is also important to promote positive self-perception of aging, as it was found that those individuals with more positive self-perceptions were more resilient during the pandemic [7].

Lastly, it is important to discuss solutions to the previously mentioned issue of increased rates of anxiety during quarantine and its associated health consequences (Table 4). The recommendation is to educate/update individuals in a precise and efficient way on the status of the virus (i.e., its consequences and spread) [6,22]. It has been amply documented that oversaturation with COVID-19 news, particularly in young people and social media users, has been linked to increased fear and anxiety [7,22,91]. As such, a constant update is needed; however, it needs to be delivered at a healthy and reasonable rate (i.e., accurate numbers, non-political bias, and reasonable time coverage) [41]. Furthermore, proper health education on face mask usage is needed to better preserve mental and physical health during the pandemic [92]. To manage the generalized anxiety about the virus and the uncertain future, support groups and services need to be established to help support individuals who are in states of uncertainty and fear (as well as those with existing psychiatric conditions) [6,22,41]. Despite our focus being on individuals in quarantine-like conditions, it is important to address that active workers, in particular front-line healthcare workers, are subject to similar deteriorations of mental well-being. Indeed, it has been reported that they have increased psychological duress, sleep deterioration, and symptoms of generalized anxiety [24,93]. While psychoneuroimmunity prevention measures are associated with a decrease in these psychological symptoms, offering peer support can further benefit the workplace [94]. It is important to understand the damage that can be imposed via a dynamic construct, such as the one presented herein (Figure 1), and the differential effects of these stress pathways on particularly vulnerable populations. In doing so, it paves the way for future research avenues which may potentially open pharmaceutical aspects to deal with severe GAD.

### 4.2. Limitations

This review has several limitations. Due to it being a narrative review, it is prone to bias (i.e., reference selection was subjective), even though steps were taken to reduce this. The primary search focus was GAD and its association with social isolation and loneliness, disregarding other mental disorders (e.g., major depressive disorder), which may contribute to our overarching construct. Future work needs to address these disorders and the roles they play, as certain individuals will have complex cases in clinical conditions. A large number of the reported effects of quarantine (for social isolation, loneliness) were obtained from online subjective questionnaires due to the quarantine, limiting the type of data that could be collected globally; these results are therefore prone to subjective bias associated with these types of questionnaires (i.e., patients using their subjective opinions to state their current mental status). Future work needs to objectively assess the consequences of the pandemic. The work used to support the mechanistic aspect of our construct (brain regions/synapses, inflammation–insulin resistance mechanism) has various limitations: the use of animal models, a small population size, and its correlational nature (as opposed to identifying causal relationships). Future work needs to address these gaps by establishing causal links for the disparate pathways involved in our proposed construct, as well as improving the previous limitations (e.g., using larger group sizes, human models, etc.). In addition, longitudinal studies are required to better establish the link between social isolation, loneliness, and symptoms of generalized anxiety in quarantine conditions. Inflammation and insulin resistance were the key biological processes focused on due to current research demonstrating their co-occurrence in various mental disorders [95]. It is important to highlight that there are other biological processes that are pertinent to GAD (e.g., the gut microbiome) [96].

Future work should analyse other biological processes that may play a role in our proposed construct. It should be noted that, to date, all loneliness scales are subjective and do not always accurately report the state of the patient. There is a need to improve patient-reported outcome scales to more accurately reflect the state of lonely and anxious patients (i.e., to better understand “how” they are feeling lonely so as to provide a more accurate response). As discussed in a recent meta-analysis by Park et al., ecological momentary assessment (EMA) has emerged as a new screening tool for loneliness; however, further research is required [13]. In terms of our proposed construct, depicted in Figure 1, the conditions mentioned (social isolation, loneliness) are risk factors, not predictive ones; an individual who is socially isolated or lonely is not guaranteed to develop symptoms of generalized anxiety. Nonetheless, the overall findings are significant as they support the possibility of our proposed construct. It may be a starting point for future research in helping explain the association of social isolation and loneliness with symptoms of generalized anxiety in quarantine-like conditions; an explanation which will be needed as this issue will arise again in future pandemics/quarantine situations.

## 5. Conclusions

The main take-away message is for health practitioners and policymakers to be aware that there will be an increase in mental health “damage” post pandemic. It is therefore important to acknowledge it, especially when a number of companies/places of employment intend employees to work from home [97]. Therefore, the question remains: how do social isolation, loneliness, and mental health disorders (such as generalized anxiety) come into play for both future employment policies and future pandemic quarantine enforcement?

## Figures and Tables

**Table 1:** The variables mentioned and their respective methods of measurement, from our referenced studies.

Outcome of Interest	Scale/Measurement
- Alcohol use	- Alcohol Use Disorder Identification Test (AUDIT) [6]
- Anxiety	- Beck Anxiety Inventory (BAI) [6]
	- COVID-19 Peritraumatic Distress Index (CPDI) [22]
	- Generalized Anxiety Disorder-7 (GAD-7) [23,24]
	- Hospital Anxiety and Depression Scale (HADS) [10]
	- Open Field Test (OFT)+Elevated Zero Maze Test (EZMT) (Mouse Model) [16]
	- Self-Rating Anxiety Scale (SAS) [8,25]
- Avoidance and compulsive behaviour	- COVID-19 Peritraumatic Distress Index (CPDI) [22]
- Cognitive change	- COVID-19 Peritraumatic Distress Index (CPDI) [22]
- Depression	- Beck Depression Inventory II (BDI-II) [6,14,23]
	- COVID-19 Peritraumatic Distress Index (CPDI) [22]
	- Forced Swim Test (FST)+Sucrose Preference Test (SPT) (Mouse Model) [16]
	- Hospital Anxiety and Depression Scale (HADS) [10]
	- Patient Health Questionnaire (PHQ-9) [24]
	- Self-rating Depression Scale (SDS) [8]
- Individual social capital	- Personal Social Capital Scale 16 (PSCI-16) [25]
- Loneliness	- ALONE Scale [26]
	- Asked three questions that allowed the authors to define “loneliness” either objectively or subjectively [27]
	- De Jong Giervald Scale [4,13,28]
	- Evaluated by means of an answer to the question: “During the last 12 months, how many times did you feel alone?” (with five choices of answers) [29]
	- Revised UCLA Loneliness Scale (R-UCLA) [14]
	- UCLA Loneliness Scale (UCLA) [4,13,30]
	- 11-point Likert Scale [7]
- Loss of social functioning	- COVID-19 Peritraumatic Distress Index (CPDI) [22]
- Mental health status	- Depression, Anxiety and Stress Scale (DASS-21) [31,32,33]
- Mental state	- Health Anxiety Inventory (HAI) [10]
- Mental wellbeing	- Warwick Edinburgh Mental Wellbeing Scale (WEMWBS) [6]
- Psychological distress	- Developed a five-item scale that measured, respectively, anxiety, anger, sadness, fear, and hope [7]
	- Psychological Distress Index (PDI) [27]
- Psychological impact of COVID-19	- Impact of Event Scale—Revised (IES-R) [31,32,33]
- Psychological profile (e.g., anxiety, well being)	- Online Ecological Recognition (OER) [9]
- Physical symptoms	- COVID-19 Peritraumatic Distress Index (CPDI) [22]
- Quality of life	- Satisfaction with Life Scale (SWLS) of Diener (1984) [34]
- RST traits	- Reinforcement Sensitivity Theory of Personality Questionnaire (RST-PQ) [23]
- Selfreport measure on health concerns	- Illness Attitudes Scale [23]
- Sleep	- Pittsburgh Sleep Quality Index (PSQI) [24,25]
- Social isolation	- ALONE Scale [26]
	- Social Isolation Scale [4]
	- Social Network Index [4]
- Specific phobias	- COVID-19 Peritraumatic Distress Index (CPDI) [22]
- Stress	- Stanford Acute Stress Reaction (SASR) [25]
- Suicide	- Time-trend regression models [35,36]
- Suicide ideation and parasuicide	- Asked three questions frequently used in health surveys (at the time) [27]
- Suicide risk	- Beck Hopelessness Scale (BHS)+Suicidal Behaviours Questionnaire—Revised (SBQ-R) [14]

**Table 2:** Physiological and psychological health issues associated with social isolation. It is important to note that many of the factors mentioned are often comorbid and associated with one another and that these consequences are health risks for “secondary” conditions (e.g., alcohol consumption is a risk factor for depression [38]).

Physiological Changes		Psychological Changes
*Body System*	*Symptom/Change*	
*Neuropsychology System*	- Panic attacks [39]	- General mental well-being deterioration [6]
	- Psychomotor excitement [39]	- Increased alcohol consumption [6,37]
*Neuromuscular System*	- Loss of muscle mass (due to sedentarism) [40]	- Loneliness [37,39,41,42]
	- Muscle damage/denervation to neuromuscular joints [40]	- Generalized anxiety [6,37,39,41,42]
*Muscle Protein Metabolism*	- Increased risk of poor metabolic health, functional decline, and all-cause mortality [40]	- Depression [6,37,39,41,42]
	- Suppression of muscle protein synthesis [40]	- Psychotic symptoms [39]
*Glucose Homeostasis*	- Skeletal muscle has a pivotal role in inactivity-induced insulin resistance [40]	- Delirium [39]
	- Specific reduction in muscle insulin sensitivity (without affecting that of the liver)	- Suicidality [37,39]
	- Insulin resistance (change in insulin sensitivity leads to muscle atrophy and change in body composition) [40]	- Symptoms of post-traumatic stress disorder (PTSD) [41]
*Cardiorespiratory System*	- Reduced cardiorespiratory fitness [40]	- Confusion [41]
	- Various steps of the oxygen pathway are impaired (e.g., central and peripheral cardiovascular function to skeletal muscle oxidative metabolism) [40]	- Boredom [41]
	- Lower/decrease in VO2max (associated with increased mortality) [40]	- Anger [37,41]
*Digestive System/Energy Balance*	- Overfeeding/comfort eating leads to systemic inflammation, weight gain, obesity, eating disorders, and muscle loss [37,40]	- Psychological conflict [23]
	- Bed rest/home isolation may be associated to decreased energy intake and rapid muscle wasting [40]	- Insomnia [25,37]
*Sympathetic Nervous System*	- Increased stress levels result in deleterious effects on cardiovascular, immune, and sleep systems [25,37,40]	

**Table 3:** Physiological and psychological health issues associated with loneliness. It is important to note that many of the issues mentioned are comorbid and associated with “secondary” conditions.

Physical Health Consequences/Risks	Mental Health Consequences/Risks
- Increased systolic blood pressure [46]	- Reduced time in bed spent asleep (7% less) (and overall sleep quality) [46,48,49]
- Increased risk of heart disease [46,47,50]	- Increased wake time after sleep onset [46,48]
- Increased risk of stroke [47,50]	- Increase in depressive symptomology [49,51]
- Vision deficits [51]	- Poor self-related health [51]
- Reduced quality of life (applies to both physical and mental aspects) [52]	- Impaired functional status/cognition [46,49,51]
- Disability (applies to both physical and mental aspects) [49,53,54]	- Perceived negative change in the quality of one’s life [49,51]
- Stress [49]	- Suicide attempts/completed suicides (among older adults) [55]
- Increased mortality [49,56,57]	- Anxiety [4,47,58,59]
- Increased use of healthcare services [49,60,61,62]	
- Institutionalization [63]	

**Table 4:** Social isolation and loneliness health implications (and risk factors) associated with generalized anxiety during the COVID-19 quarantine.

Direct	Indirect
- Anxiety [6,8,22,25,37,39,47,67,68]	- Alcohol consumption [6,37]
- Panic attacks [22,39]	- Depression [6,8,22,37,39,42,47,67,68]
- Delirium [39]	- Boredom, anger [39,67]
- Fear, distress, general stress [7,22,25,37,47,67,68]	- Suicidality [37,39,67,68]
- Insomnia [7,25,37,68]	- COVID-19/mortality fear [7,22,37,42,67]
- Higher all-cause mortality (both independent risk factors) [46]	- Running out of life-sustaining medical supplies/care/access [8,22,42,46]
- Incident dementia [46]	- Oversaturation to COVID-19 news (via radio, TV, social media) [7,22,42]
- Low self-perceived health condition [8]	- Increased risk of coronary artery disease-associated death (even with no prior history) [46]
- Unemployment/economic loss [8,47,68]	- Cardiovascular disease [47]
- Previous psychiatric history (recurrent or induced) [10,46]	- Chronic health illnesses [10]

## Data Availability

Not applicable.
